# Multidimensional Profiling of Human Body Hairs Using Qualitative and Semi-Quantitative Approaches with SR-XRF, ATR-FTIR, DSC, and SEM-EDX

**DOI:** 10.3390/ijms24044166

**Published:** 2023-02-19

**Authors:** Karen J. Cloete, Žiga Šmit, Alessandra Gianoncelli

**Affiliations:** 1 UNESCO-UNISA Africa Chair in Nanosciences and Nanotechnology Laboratories, College of Graduate Studies, University of South Africa, Muckleneuk Ridge, P.O. Box 392, Pretoria 0003, South Africa; 2 Nanosciences African Network (NANOAFNET), iThemba LABS-National Research Foundation, P.O. Box 722, Somerset West 7129, South Africa; 3 Faculty of Mathematics and Physics, University of Ljubljana, Jadranska 19, SI-1000 Ljubljana, Slovenia; 4 Jožef Stefan Institute, Jamova 39, SI-1001 Ljubljana, Slovenia; 5 Elettra Sincrotrone Trieste, Strada Statale 14, km 163.5 in Area Science Park, 34149 Basovizza, TS, Italy

**Keywords:** ATR-FTIR, DSC, EDX, elemental micro-mapping, body hair, SEM, SR-XRF

## Abstract

This study aimed to assess the potential of a multidimensional approach to differentiate body hairs based on their physico-chemical properties and whether body hairs can replace the use of scalp hair in studies linked to forensic and systemic intoxication. This is the first case report controlling for confounding variables to explore the utility of multidimensional profiling of body hair using synchrotron synchrotron microbeam X-ray fluorescence (SR-XRF) for longitudinal and hair morphological region mapping) and benchtop methods, including attenuated total reflectance Fourier transform infrared spectroscopy (ATR-FTIR) (complemented with chemometrics analysis), energy dispersive X-ray analysis (EDX) (complemented with heatmap analysis), differential scanning calorimetry (DSC), and scanning electron microscopy (SEM) analysis (complemented by descriptive statistics) to profile different body hairs in terms of their elemental, biochemical, thermal, and cuticle properties. This multidimensional approach provided supportive information to emphasize the intricate and rather complex interplay between the organization and levels of elements and biomolecules within the crystalline and amorphous matrix of different body hairs responsible for the differences in physico-chemical properties between body hairs that are predominantly affected by the growth rate, follicle or apocrine gland activity, and external factors such as cosmetic use and exposure to environmental xenobiotics. The data from this study may have important implications for forensic science, toxicology and systemic intoxication, or other studies involving hair as a research matrix.

## 1. Introduction

Hair is one of the most interesting biological matrices applied as a screening matrix within the field of biomedicine, forensic science and toxicology to screen exposure to xenobiotics such as toxic metals and other elements due to its unique morpho-chemical properties and a range of other benefits [1,2]. First, hair may be the only trace evidence that is resistant to decay due to its high keratin content [3]. Second, non-invasive hair sampling is possible under close supervision, whilst hair samples can also be stored for long-term periods and transported with ease [1,4]. Most important, hair concentrates and stores chemical information contained within the circulatory system and, depending on its length, provides a retrospective or chronological profile of acute or chronic exposure to xenobiotics, including toxic metals [1,4].

Although the literature supports the use of hair as a screening tool in various fields, its routine use to provide credible data is still heavily debated, mainly due to the many confounding factors that may significantly skew chemical data extracted from scalp hair which is most commonly sourced for analysis [1,5]. It is also possible that the hair chemical profile, which is interlinked with hair morphology and material, including thermal properties [6,7], may also significantly differ between scalp hair regions and hair retrieved from different body parts. Unfortunately, there are currently no reports focused on the physico-chemical profile in virgin hair fibers representing body hairs and controlling for inter-individual confounding variables in terms of hair treatments, hair color, age, gender, diet, smoking habits, general health status, medication use, and sampling season [1].

Hair is a complex tissue that contains relatively low levels of chemical elements apart from carbon (C), hydrogen (H), oxygen (O), and nitrogen (N) [2,7,8] and hence requires accurate and sensitive analytical techniques for chemical elemental profiling and distribution mapping. Synchrotron microbeam X-ray fluorescence (SR-XRF) remains one of the most versatile analytical techniques with excellent detection selectivity, sensitivity, and spatial resolution for elemental micro-mapping [9]. Intact samples can be non-destructively analyzed without the need for chemical fixation, dehydration, or electroconductive coating that may introduce artefacts, lead to elemental losses, or destroy historical and spatial information contained along the length of a hair fiber [10]. Most importantly, SR-XRF analysis allows for assessing the time-resolved evolution of elemental exposure via longitudinal mapping along the hair length [11,12]. Combined with elemental analytical methods such as energy dispersive X-ray analysis (EDX) linked to scanning electron microscopy (SEM), additional information can be obtained from a semi-quantitative perspective from both an elemental and hair morphological point of view.

Another micro-spectrometry technique that presents as a powerful tool in non-destructive chemical analysis is Attenuated Total Reflectance Fourier Transform Infrared Spectroscopy (ATR-FTIR) [13]. FTIR is an ambient temperature analytical method used to determine infrared absorption due to the energy resonance of vibrational motions of chemical bonds within functional groups that also characterize molecular structure [14]. FTIR has better sensitivity, higher resolving power and wavelength reproducibility, and almost no interference from fluorescence compared to Raman spectroscopy—another popular technique in hair analysis—that may lead to sample damage due to the prolonged exposure of the specimen to a laser beam to achieve an enhanced Raman spectrum of the sample [15]. More specifically, ATR-FTIR spectroscopy is based on the occurrence of total internal reflection in which infrared radiation (IR) is directed into an internal reflection element (a high refractive index crystalline material), the sample absorbs IR at certain frequencies, and the consequent totally reflected radiation is either attenuated or altered in regions of the infrared spectrum where the sample absorbs energy [16]. ATR-FTIR avoids excessive absorbance of IR translating into minimization of peak or band saturation of, i.e., the Amide I and Amide II bands and is further insensitive to sample thickness [17]. ATR-FTIR can hence present as a complementary tool for multidimensional hair chemical characterization and screening by providing information on hair chemical functional groups at the molecular level in supplement to SR-XRF and EDX that provide elemental mapping and concentration data across the longitudinal hair length, representing chronological exposure, and cross-sectionally, representing extrinsic versus intrinsic exposure.

As the chemical, as well as morphological properties of hair can also influence its material properties [18], material analysis of hair with techniques such as differential scanning calorimetry (DSC) that measure thermal conductivity has received increasing interest, especially within the fields of hair cosmetics and dermatology to study changes in hair morphology and chemistry induced by hair treatments [19]. As hair is a biological material, its inherent thermal properties defined by its chemistry and morphology may hence present as a potential additional complementary and differential analytical tool in hair analysis. Unfortunately, hair thermal analysis has not yet been fully explored in, for example, hair forensic analysis.

Here, multidimensional profiling using qualitative and semi-quantitative approaches was used to characterize body hair samples retrieved from one individual and control for inter-individual hair confounding variables [1]. Low energy SR-XRF was used to (1) determine longitudinal elemental distribution in intact body hair fibers and (2) determine the distribution of elements in body hair cross-sections representing different hair morphological regions to distinguish external from endogenous exposure to chemicals. The hair selected for this study included eyebrow, underarm, pubic, leg, and scalp hair. Low energy SR-XRF was also complemented with EDX combined with heatmap analysis, ATR-FTIR supported with chemometrics analysis, DSC, and SEM analysis supported with descriptive statistics to differentiate body hairs based on their elemental, biochemical, cuticular morphometry, and thermal properties. This study aimed to answer the following questions: (1) do body hair differ in their physico-chemical properties?; (2) do cross-sectional and longitudinal elemental mapping of different body hair types advising intrinsic and extrinsic exposure present differential profiles between body hair types?; (3) based on the aforementioned findings, which hair can provide supportive and non-conflicting data if scalp hair cannot be sampled due to alopecia or other factors?; and (4) could analysis of hair using complementary and advanced synchrotron and benchtop techniques provide a novel multidimensional approach to hair analysis?

## 2. Results and Discussion

### 2.1. SR-XRF

**Figure 1:**
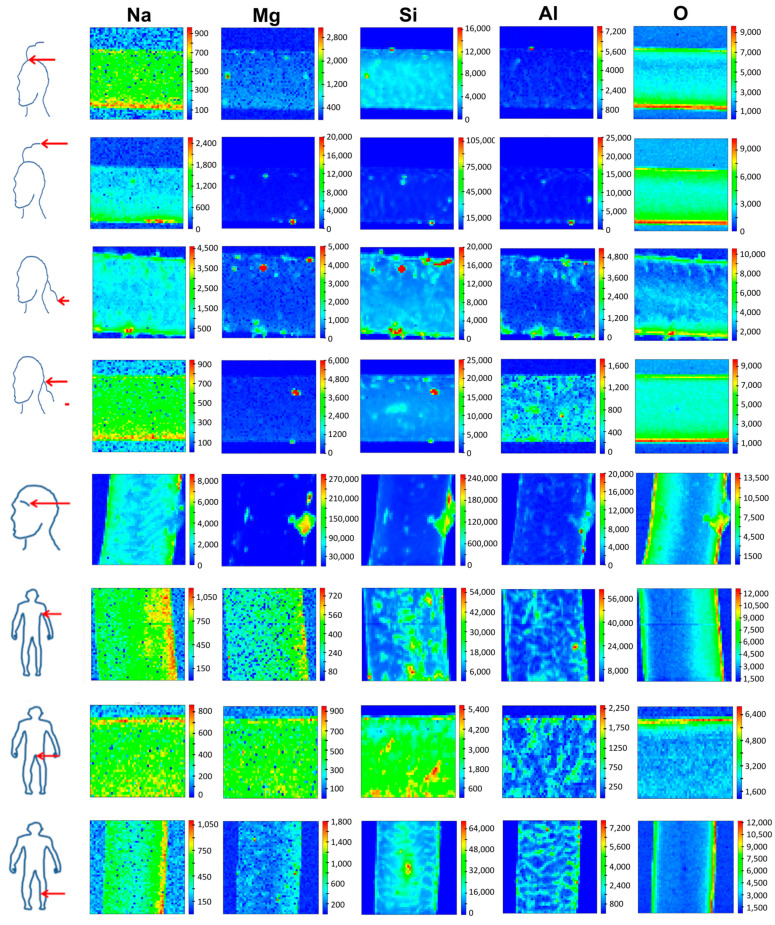
Synchrotron microbeam X-ray fluorescence mapping of longitudinal hair sections from different body parts (scalp regions—left panel, and eyebrow, underarm, pubic, and leg hair—right panel) donated by the same person showing the elemental distribution of sodium, magnesium, silicon, aluminium, and oxygen. Elemental maps, all 80 µm × 80 µm in size, were produced with a 500 nm X-ray beam spot size to compromise between the signal and size of the features of interest. The detected elements increase from the blue color to the red color in the scale bar, with the colors indicating relative element content.

**Figure 2:**
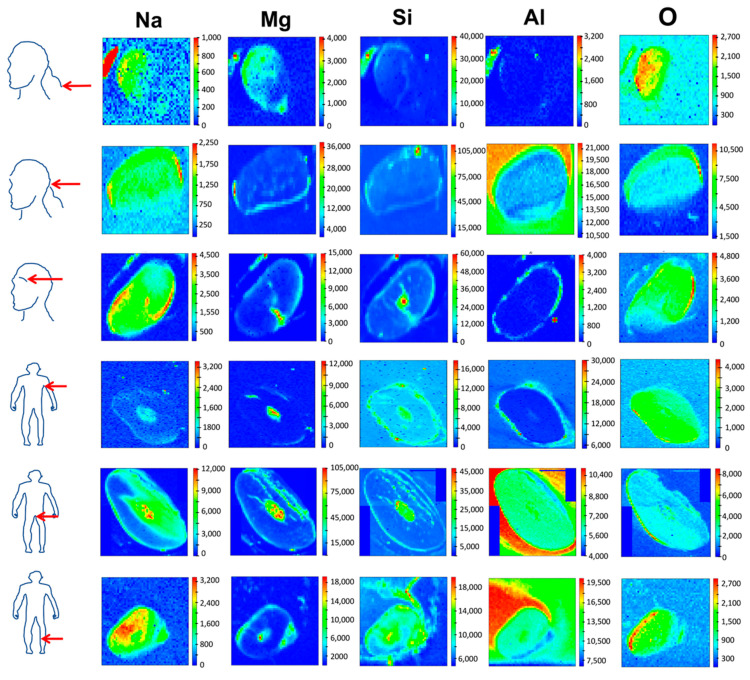
Synchrotron microbeam X-ray fluorescence mapping of hair cross-sections originating from different body parts (scalp regions, eyebrow, underarm, pubic, and leg hair) donated by the same person sectioned with a microtome to a thickness of 10 µm (light micrographs of hair cross sections). The elemental distribution of the detected elements is shown across the different hair morphological regions. Elemental maps were, all 80 µm × 80 µm in size, produced with a 500 nm X-ray beam spot size to compromise between the signal and size of the features of interest. The detected elements increase from the blue color to the red color in the scale bar, with the colors indicating relative element content.

This is the first report utilizing SR-XRF to compare body hair’s exogenous and endogenous elemental composition. SR-XRF is a spatially resolved and relatively rapid method, and its application to analyze complex tissues such as hair to determine heterogeneous elemental distributions on a spatial scale hence presents promising applications in research utilizing hair as a matrix [9]. The TwinMic X-ray spectromicroscopy beamline at the Elettra synchrotron proved to be a powerful tool to provide spatially resolved and non-destructive qualitative elemental mapping of body hairs across its longitudinal profile (Figure 1). Since no standardized washing protocol for hair exists and washing may also not remove elements strongly bound to the hair cuticle scales, unwashed hair was analyzed shortly after sampling [1]. SR-XRF also allowed cross-sectional mapping of hair (Figure 2) to discriminate between xenobiotic elements from external contaminants and pollutants that may either be trapped in the cuticle scales or diffuse into the hair fiber or endobiotic elements deposited by blood into the hair’s internal regions, i.e., medulla and cortex [1,2]. To cross-section hair, wax was used as an embedding medium as it was previously reported that embedding in the resin might alter the elemental hair fingerprint due to the desolation of weak electrostatic bonds between elements such as metals and the hair cuticle in contact with the resin [20].

Sodium (Na) detected in the hair samples may originate from the use of cosmetic products such as shampoo, where it is used as a thickener, the consumption of highly processed food, water source, or sweat which contains higher amounts of Na than magnesium (Mg) [21,22,23]. Reports have also shown that significant differences were observed between the apocrine and eccrine glands in terms of Na^+^ secretions [24], which may also explain the difference in the distribution and intensity profile for Na and Mg across the different body hairs and the co-localization of Mg and Na in pubic and underarm hair that may be more exposed to bodily secretions such as sweat. Magnesium and silicon (Si) may also originate from cosmetic products [25], whilst the water source exposed may also contribute to the levels of Mg, Si, and aluminium (Al) in hair [26]. Aluminium however may originate from a wide diversity of anthropogenic or geogenic sources, i.e., water treatment chemicals and water pipes, cooking utensils, personal care products, vaccination, and some drugs and antacids [26,27].

Figure 1 shows Na, Mg, Si, Al, and O distribution mapped across the longitudinal profile of scalp, eyebrow, underarm, pubic, and leg hairs. For Na, the distribution pattern and intensity were similar between scalp hair retrieved at the top of the head, nape of the neck, leg, pubic, and underarm hair. On the contrary, scalp hair ends sampled from the top of the head and nape of the neck area as well as eyebrow hair, showed a different Na distribution pattern and higher intensity. The distribution pattern and intensity of Mg and Si also differed between different body hairs, although Mg and Na seemed to co-localize well in pubic and underarm hair, where it appears more uniform, while in other body hairs, Mg is present also as hot spots. Interestingly, the distribution and intensity of Al were similar amongst scalp hair from the nape of the neck area, underarm, pubic, and leg hair. In all other body hairs, Al also appears as hot spots, sometimes co-localizing with Si and Mg. For O, hair ends in the nape of the neck area, and pubic hair shared a similar distribution pattern. The heterogeneity in elemental distribution and intensity for longitudinal and cross-sectioned hair can be ascribed to many different factors. For example, the location of the body hair, its physico-chemical makeup, as well as exposure to external contaminants or pollutants, sweat and sebum, personal care products or cosmetic treatments, and ultraviolet (UV) light [1]. Indeed, underarm and pubic hairs seem to have fewer Al-Mg-Si hot spots. The effect of diet, smoking habits, general health, occupation and economic status, cultural practices, nationality, gender, age, personal habits, and environmental factors, i.e., seasonal fluctuation, urban vs rural residence, and distance from pollutant sources [1] is negligible in this study as contributory factors as only one individual’s hair was sampled. Depending on hair length, longitudinal mapping has been shown to be a valuable tool to study the incorporation of elements or metabolized compounds over time as illustrative of a case of lead pollution in a lead smelter worker [11] and Phar Lap—the famous racehorse, with a longitudinal change in the intensity signal with time progression linked to metabolic changes incorporated during hair formation [28]. However, as shown by the current study, intensity patterns may differ depending on where the hair is sampled on the body, with different hairs also exhibiting different growth metabolism and follicular activities [1].

Since the nape and top of the head hair had similar distribution patterns, cross-sectional analysis was performed on the nape of the neck hair, which is also often used as an area for hair sampling [29]. In cross-sectioned hair (Figure 2), scalp hair sampled from the nape of the neck area and eyebrow hair had higher intensities of Na in the cuticle region and also for the medulla in eyebrow hair. Pubic and underarm hair had higher intensities of Na in the medulla, whilst leg hair had higher Na intensities in the hair cortical regions. For Mg and Si, higher intensities were observed in the cuticle except for underarm, pubic, and leg hairs in which Mg was concentrated in the medulla, and eyebrow, pubic, and leg hairs in which Si was concentrated in the internal hair regions. For Al, higher intensities were observed in the cuticle scales for scalp, eyebrow, and underarm hair, whilst pubic and leg hair showed higher intensities in the hair internal regions, confirming the uniform distribution seen in the longitudinal mapping. For O, differential distribution patterns were noted between the different body hairs, although leg, underarm, and eyebrow hair showed an almost similar distribution pattern across the hair morphological regions. Overall, the elements detected showed higher intensities within the hair’s internal regions, except for underarm hair (Na), scalp and eyebrow hair (Mg), scalp hair (Si), and eyebrow and underarm hair (Al). Besides longitudinal analysis, the cross-sectional analysis also showed differential distribution patterns between different body hairs, which may be ascribed to their unique physico-chemical properties and other external factors, including the differential exposure to sweat and personal care products, as mentioned in the previous paragraph [1]. The differential elemental distribution or intensity patterns within the different body hairs analyzed could also be ascribed to the binding of positively charged elements supplied via the bloodstream to hair pigment granules or melanosomes within the hair internal regions of more heavily pigmented hairs [30,31]. Although the exact mechanism of elemental incorporation into hair is unclear, it has been proposed that elements may enter the hair via active or passive diffusion from the bloodstream, diffusion from sweat or other excretions, or via external diffusion from the air [1].

### 2.2. EDX

**Figure 3:**
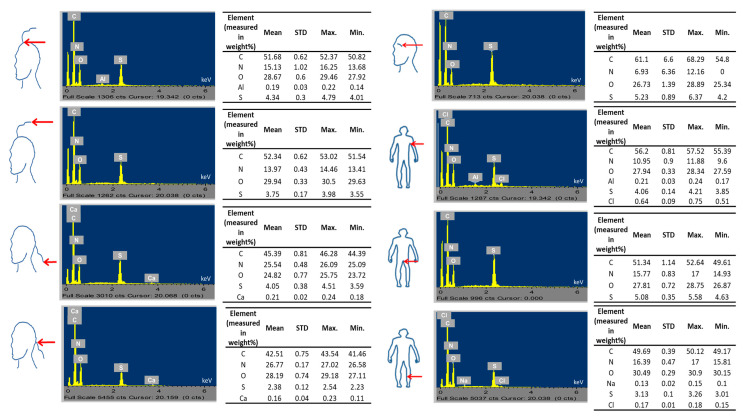
Energy-dispersive X-ray spectroscopy data showing the spectra and mean, standard deviation, maximum, and minimum values for elements of exogenous (aluminium, chlorine, calcium, and sodium) and endogenous origin (carbon, nitrogen, oxygen, sulphur) detected in body hair (scalp regions, eyebrow, underarm, pubic, and leg area) donated by the same person.

Besides qualitative multi-elemental imaging and mapping using SR-XRF of longitudinal and cross-sectioned body hairs, semi-quantitative analysis (in weight percentage) of essential hair elements, including C, N, O, and sulphur (S) in the hair surface tissues was also performed with SEM-EDX. The elemental data showed that the highest proportion was found for carbon, followed by N, O, and S, which is expected for the proportions of endogenous elements in hair forming part of hair proteins, lipids, and other hair biochemical constituents (Figure 3) [7]. However, carbon may also originate from carbon coating the sample prior to analysis, whilst S may also originate from hair cosmetics [32]. More specifically, carbon % ranged from 42.51 to 61.1%, nitrogen from 6.93 to 26.27%, O from 24.82 to 30.49%, and S from 2.38 to 5.23%, with the variability of N among body parts being higher than the variability of O. Based on descriptive statistical analysis, the mean data from highest to lowest values for hair elements was as follows: C (eyebrow > underarm > top of scalp end > top of scalp > pubic > leg > nape of neck > nape of neck hair end); N (nape of neck area > leg > pubic > top head area > underarm > eyebrow); O (leg > top of head hair end > top of head > nape of neck area > underarm > pubic > eyebrow > nape of neck hair end); and S (eyebrow > pubic hair > top of head > underarm > nape of neck > top of head end > leg > nape of neck hair end) (Figure 3). Mean and standard deviation, as well as maximum and minimum values, are outlined in Figure 3. Other than the endogenous elements, some exogenous elements such as Al (underarm and scalp hair on top of the head), calcium (Ca) (scalp hair from the nape of the neck), and chlorine (Cl) (leg and underarm hair) were also detected (Figure 3).

Secondary statistical analysis with hierarchical clustering showed the similarities and differences in the endogenous elemental composition of the different body hairs based on location and the similarity or differences between locations based on hair endogenous elemental content. The color legend of the heatmap (Figure S1) indicates a lower average elemental concentration (blue) or a higher average elemental concentration (red) in a particular sample. Hair at the nape of the neck hair showed above average z-scores and top head hair ends below average z-scores. The highest dissimilarity to average values (extreme z values as shown by the brightest colors of the color map) was observed for the z-scores of leg and eyebrow hair. Interestingly, the cluster analysis of the hair regions provided a dendrogram clustering eyebrow, and underarm hair, hair on the top of the head and pubic hair, nape of the neck hair, and hair ends at the top of the head with leg hair, with the latter group separated from all other body hair groups. The cluster analysis of the elements showed two large clusters. The first smaller cluster grouped C and S, which formed a larger cluster with N, and with C, S, and N forming another bigger cluster with O. In sum, leg hair showed a strong positive signal for O and a strong signal in the opposite direction for N and C; top of the head hair ends a strong negative signal for S; underarm hair a strong negative signal for O and eyebrow hair, strong positive signals for C and S; and finally, nape of the neck hair showed a strong positive signal for N. The clustering of these endogenous hair elements hence indicates the unique chemical makeup of the different body hairs influenced by various external and internal factors [1]. 

As there are no studies available on comparing different body hairs with SR-XRF and EDX and based on the unique physico-chemical profile of different body hairs linked to a specific individual [1], we cannot compare the results of this report with available published data. Nevertheless, SR-XRF and EDX proved to provide very useful chemical fingerprinting tools for delivering qualitative longitudinal and cross-sectional hair morphological mapping as well as semi-quantitative assessment to differentiate exogenous versus endogenous elemental profiles. Furthermore, EDX was also able to detect the endogenous elements C forming part of hair lipids, S and N forming part of hair amino acids and proteins, and O forming part of other hair biochemical constituents [33]. Most importantly, SR-XRF and EDX spectroscopies allowed non-destructive and rapid analysis compared to wet chemical methods such as inductively coupled plasma atomic emission spectroscopy, inductively coupled plasma mass spectrometry, and atomic absorption spectrometry [34].

### 2.3. ATR-FTIR

**Figure 4:**
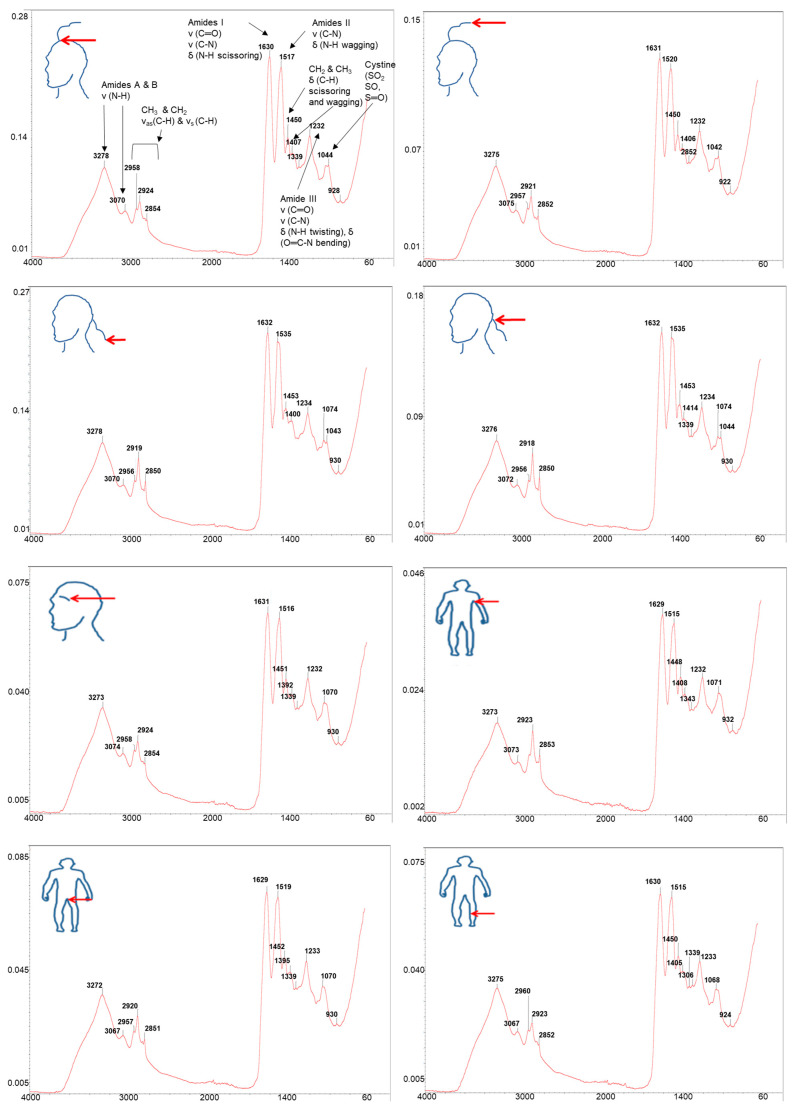
Attenuated Total Reflectance Fourier Transformed Infrared Spectroscopy spectra for hair from different body parts (scalp region, eyebrow, underarm, pubic, and leg areas) donated by the same person. The spectrum produced displays the intensity (energy absorbed) as a function of wave frequency (wavenumber in cm^−1^).

Figure 4 shows ATR-FTIR spectra with marked wavenumbers as separate figures for comparison purposes of the different body hairs collected with a resolution of 4 cm^−1^ at 25 ℃ and within the spectral range of 4000–60 cm^−1^. Only baseline correction was applied, and no other spectral treatments were performed on the spectra. The spectra were typical of hair which is primarily comprised of biomolecules such as alpha keratin, related proteins, and lipids, amongst other compounds [35]. A broad band is observed at 3278 cm^−1^, which could be attributed to the O−H stretching of water together with an N−H stretching vibration [35,36]. The presence of Amide I vibration at 1630 cm^−1^, Amide II at 1516 cm^−1^, and Amide III at 1231 cm^−1^ are further confirmed [35,36]. The Amide I vibration corresponds to C=O stretching vibration and a small contribution from N−H scissoring vibration, generally pointing to a well-preserved cuticle vibration [35,36]. The absorption band of Amide II consists of the C−N stretching and N−H wagging vibrations, whilst the Amide III absorption band consists of the N−H twisting vibration, C−N stretching vibration, and the contribution from O=C−N bending vibration [35,36]. In addition, the absorbance band at 930 cm^−1^ could be associated with the vibration O=C-N for Amide IV [35,36]. The methyl (CH_3_) asymmetric stretching absorption band at 2957 cm^−1^ and symmetric stretching mode at 2924 cm^−1^ were further observed, as well as the methylene (CH_2_) symmetric stretching mode absorption band at 2853 cm^−1^ (C−H stretching usually assigned to lipids) vibration, whilst the absorption band at 1338 cm^−1^ represents the CH_3_ bending deformation vibration of an amino acid vibration [35,36]. Other bands represent the absorption bands of keratin proteins (3070 cm^−1^) and cystine (1044 cm^−1^) which contain disulphide bonds with high S levels responsible for the cross-linking of peptide chains within the hair vibration [35,36].

**Figure 5:**
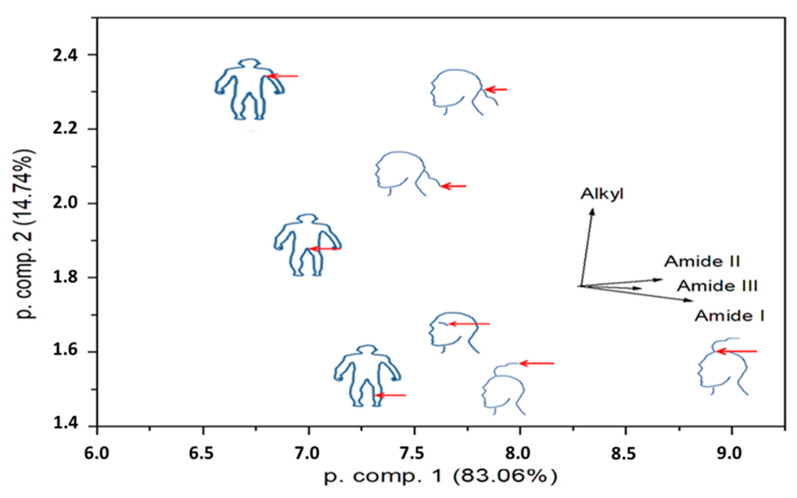
Principal component analysis of the Attenuated Total Reflectance Fourier Transformed Infrared Spectroscopy data.

The spectra of the different body hairs did not exhibit large differences, although some variation in peak shape and position particularly around the cystine absorption bands were noted, pointing to different structural conformations of biomolecules between different body hairs. For quantitative analysis of the spectra (Figure 4), intensities were determined for the Amide I-III and Alkyl lines from the individual ATR-FTIR spectra. After the linear background below the peak was subtracted, the obtained intensities were normalized according to the total counts in the spectrum. For the control, the peak values of H_2_O peaks were also determined and normalized to the total spectral counts. The respective ratios had a standard deviation of 6.9%. The deduced intensities were next regarded as input data for principal component analysis (PCA), with an aim to reduce the four intensities for a display in two dimensions. No transformation of the input data was completed. The results for the first two principal components show that the eigenvectors of all Amide intensities are pointing almost in the same direction, while the eigenvector of Alkyl intensities is roughly perpendicular to them (Figure 5). This means that the samples do not distinguish according to the relative Amide content but rather according to the relative proportion of Amide and Alkyl groups.

In sum, ATR-FTIR analysis can provide rapid, label-free, and non-destructive analysis of samples that require minimal sample processing to provide specific chemical information of functional groups in the sample at the molecular level [35,36,37]. Although no reports exist combining ATR-FTIR, SR-XRF, and SEM-EDX analysis of hair samples, ATR-FTIR very well complements SR-XRF and EDX measurements to provide biochemical information at the molecular level. Furthermore, although the technique has gained popularity in forensic and cosmetic sciences, it has not yet been explored to differentiate between different body hairs. When combined with an omics-based discriminatory tool, such as a chemometrics approach using PCA analysis, hair samples can also be more readily differentiated [38]. Although we are not aware of any studies combining ATR-FTIR with chemometrics to differentiate body hairs, a recent study by Manheim and coworkers [39] used ATR-FTIR spectroscopy combined with chemometrics to successfully and reliably classify and discriminate between hair retrieved from humans, cats, and dogs. In essence, such differences between hair samples are underscored by differences in the hair biochemical fingerprint due to hair damage (induced by ultraviolet irradiation, heating, and brushing), cosmetic treatment, diet, and health status, amongst other factors, which also affect the hair’s ability to absorb exogenous and endogenous elements as well as affects its material including thermal properties [1].

### 2.4. DSC

**Figure 6:**
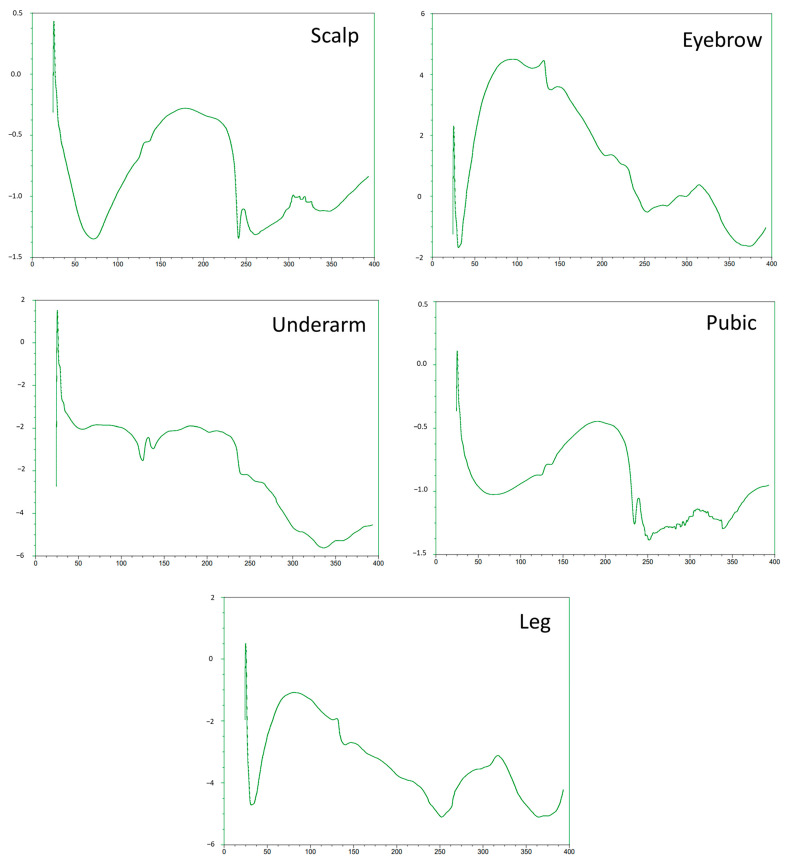
Differential scanning calorimetry (DSC) curves from the different body hairs (scalp area, eyebrow, underarm, pubic, and leg areas) donated by the same person. DSC was carried out using a TA Instruments Q200 instrument, and the temperature ramped between 253 and 573 K using a rate of 278–283 K min^−1^ depending on the sample. Dry nitrogen gas was used to purge the furnace at a flow rate of 50 mL min^−1^. X-axis on all figures: Heatflow (W/g); Y-axis on all figures: Temperature (℃).

To screen the ability of thermal analysis as a potential screening tool to complement the forensic analysis of body hair types, dry-DSC was used to screen differences in the thermal behavior of body terminal and intermediate hair. Here we note the DSC curves (Figure 6) for the untreated and virgin scalp, eyebrow, underarm, pubic, and leg hair retrieved from one individual with known information on age, gender, diet, medical and medication history, hair treatment, and personal habits. The curves show unique patterns in terms of curves and shapes between the different body hair types, with leg and eyebrow, as well as scalp and pubic hair showing fairly similar patterns in couples. However, underarm hair shows a dissimilar pattern compared to other body hair types. The curves further show several thermal events between 30 and 400 ℃, with an endothermic event occurring between 30 and 60 ℃ and with a minimal temperature varying between 30 and 40 ℃ for eyebrow and leg hair and 60–70 ℃ for scalp hair. This minimal temperature could be attributed to the elimination of loosely bound water from the capillary tissue [40,41]. Interestingly, these endothermic peaks were broader for leg hair and sharper for eyebrow hair, which could be ascribed to orientational inhomogeneities, heterogeneous crystal size distribution, or differing water levels and water bonding between these hairs [40,41]. Between 140 ℃ and 200 ℃, a constant slope of heat flow was observed, pointing to changes within the glass transition region of amorphous and cysteine-rich keratin of high quality and loss of more strongly bound water [40,41]. Other endothermic peaks were observed at 135 ℃ for leg, and eyebrow hair and a doubled peak around 240 ℃ for pubic hair. These peaks could be attributed to the thermally induced breaking of chemical bonds (cystine disulfide linkages) of the crystalline alpha-helices forming the ordered keratin intermediate filaments or microfibrils exhibiting low S content and stabilized by water, or to the thermal denaturation of the high S cystine content of the amorphous matrix (second doubled peak associated with pubic hair) [40,41]. No other endothermic peaks were observed below 250 ℃ for the leg and eyebrow. Another isotherm peak (exothermic or onset of endothermic) was observed above 300 ℃ for leg and eyebrow hair, indicating oxidation (e.g., amide cross-linking), decomposition, or pyrolysis of hair organic matter [40,41].

Hair is a rather unique and very complex biological material featuring unique physico-chemical properties at the ultrastructural and microstructural levels that may also influence its thermal properties [41,42,43]. Although the thermal properties of body hair have not yet been investigated, these recorded thermal events between different body hairs from one individual may point to the complex interplay, organization, and levels of biomolecules (including melanin and water) within the crystalline and amorphous matrix of different hairs that also contribute to their structural integrity and strength, factors that are further vulnerable to chemical (including the introduction of xenobiotics such as metals) as well as mechanical (combing and curling), and most importantly, thermal treatments (hair drying and straightening) [40,41,43] that may differ between different body hairs. Past research has, for example, screened the thermal behavior of hair retrieved from students and officials representing different ethnic groups using DSC, although at higher temperatures (up to 600 °C) than our study. The data showed that although hair DSC curves did not possess enough discriminative power for ethnic classification, the data did show unique characteristics linked to a specific individual [44]. Furthermore, other studies would include analysis of the thermal properties of the DSC plots, such as onset temperature, melting temperature, and melting enthalpy [40,41], although, in this study, we were not interested in quantitatively studying melting dynamics of alpha-keratin between different hairs for which DSC finds unique applications in cosmetic hair studies related to hair damage and repair. This preliminary data, however, shows that thermal analysis with DSC may hold unique potential in forensic hair analysis at the micro-level linked to hair structure and the environment enveloping microscopic and molecular structures that may be influenced by a range of factors. However, careful interpretation and understanding of hair chemistry and structure are warranted to produce a reliable interpretation of data obtained using DSC analysis.

### 2.5. SEM

**Figure 7:**
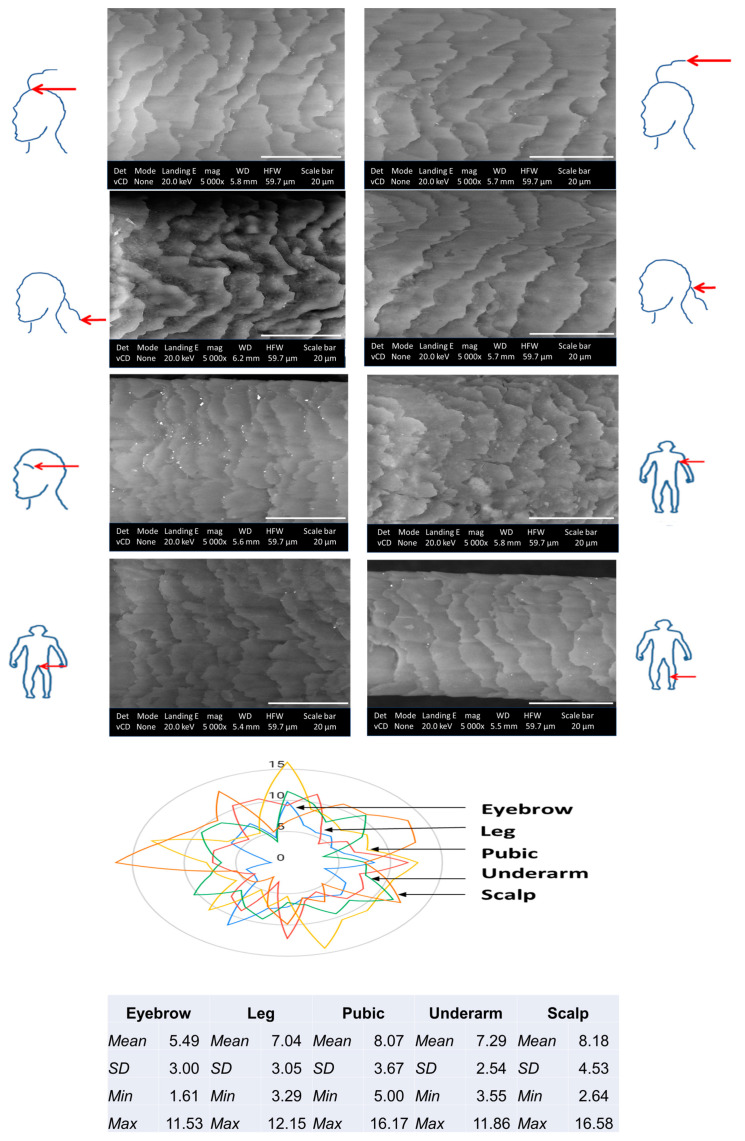
Scanning electron microscope (20 keV, ×5000) images showing the morphology of the cuticle scales of different body hairs (scalp region, eyebrow, underarm, pubic, and leg areas) donated by the same person. Scale bar: 20 µm. Descriptive statistics of scale layer differences (mean value of ca. ten measurements on a single strand of hair) are presented in the table and spider graph.

Figure 7 shows SEM micrographs of different body hair types and data from morphometric measurements focused on scale layer differences between body hairs. Based on qualitative inspection focused on the scale margins, shape, and distance, these hair types all show imbricate cuticle scale patterns or flattened and overlapping scales with narrow to intermediate-distant as well as crenate-type and irregularly wave-shaped margins [45]. For some hairs, i.e., scalp hairs, smoother cuticle margins were visible. Interestingly it has been reported that the scale characteristics may change with hair length [46]. Here, similar data was observed as the tip of hair samples at the nape of the neck area showed slightly more raised cuticle scales which could be due to mechanically induced hair damage (brushing and exposure to UV rays) or changes to the surface morphology of the cuticles as a consequence of chemical compositional changes, i.e., a lower content in cystine linked to S levels, therefore, fewer disulfide bridges in the A-layer [7]. Interestingly, SEM-EDX data also showed the presence of lower S content in the hair ends, which may explain the more raised cuticle scales at the hair ends.

As hair morphological comparison analysis is subjective, the morphometric features related to cuticle scale layer differences were also assessed using computer software and represent the average value of ca. ten distances between two adjacent scales on the same hair. Interestingly, hair scale layer differences retrieved from different regions and distance from the scalp showed mean differences in micrometers (Head hair ends:10.7 ± 2.9 > Nape of neck hair ends 9.5 ± 3.2 > Nape of neck: 8.5 ± 3.3 > Head 8.3 ± 2.8). Scalp, pubic, and underarm hair showed higher cuticle scale layer differences than the rest of the body hair sampled, whilst the mean values for leg and eyebrow hair were almost similar (Figure 7). The variation in the cuticle scale layer differences between different body hairs could likely be attributed to the hair cycle that varies between hair from different body regions [47,48]. For example, scalp hair has a longer growth cycle than other body hairs. 

SEM is presented as a very attractive technique for non-destructive and high-resolution imaging of the hair cuticle scale ultrastructure for morphometric analysis using computer software. Although morphometric analysis is often used in wildlife forensic studies for species differentiation and identification by assessing hair cuticle scale morphological characteristics such as cuticle scale patterns, the margin type, as well as shape and distance between scales [49,50,51], morphometric profiling of different body hair cuticle scales has never been investigated. We are only aware of one study that focused on studying the morphology of different body hairs in a male participant [52] and one that assessed cuticle scale morphometrics and showed differences based on gender and sampling region on the head, similar to our study [53]. Other reports have mostly focused on discriminating body hairs based on characteristic length, color, shape, root appearance and internal microscopic analysis of the medulla characteristics, cortical texture, thickness, and diameter, as well as pigment density and distribution [54]. Based on the results of this study, it is highly probable that cuticle scale margin morphology and cuticle scale layer differences between human body hairs may also present as an additional hair discriminatory feature besides hair chemical makeup, further supported by the fact that hair morphometry, e.g., hair shaft width in both animals and humans have been found to be greatly influenced by differential metabolic and nutritional states between individuals [51].

**Figure 8:**
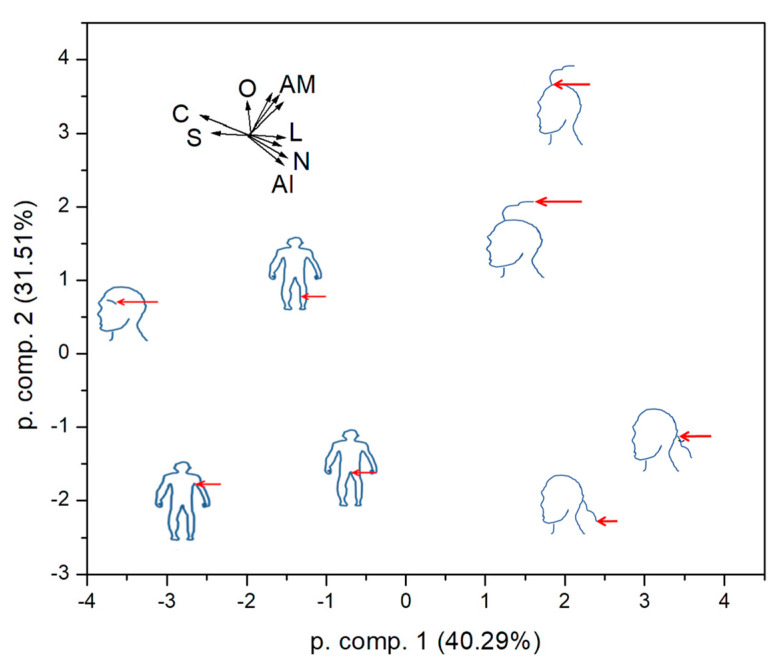
Principal component analysis of the studied hair according to the combined chemical and geometrical parameters. C, N, O, S: elemental concentrations; L: mean scale length and standard deviation, AM: amide peaks; Al: alkyl peak.

Finally, we tried to classify the samples according to all physical parameters that could be quantified. We constructed a set of ten-dimensional property spaces. The first four dimensions were mean concentrations of C, N, O and S, as obtained by EDX. The fifth and sixth dimensions were the mean scale length and its standard deviation as measured in SEM. The last four dimensions were the normalized intensities of the three amide and alkyl peaks in the FTIR spectra. We could not evolve any well-defined parameters from the DSC and SR-XRF data. As the obtained quantities represent quite different physical parameters, the data were transformed to zero mean and unit standard deviation before the PCA calculation. The first two components retain 71.8% of the original variation (Figure 8). The plot shows that hair close to the scalp and hair ends in the nape of the neck and scalp area are similar. This is expected and gives credibility to our procedure. One can deduce that underarm and pubic hair also bear similarity, while eyebrow is specific, lying in the direction of high C and S concentrations.

## 3. Methods and Materials

### 3.1. Collection of Hair Samples

This was a case study in which virgin body hair (*n* = 24) that has not been subjected to cosmetic treatments was randomly sampled during the winter season within each of the different body regions (scalp, eyebrow, underarm, pubic, and leg regions) from one female individual (age 39) to control for inter-individual variability in hair chemical makeup and other confounding variables including age, gender, cosmetic treatments, smoking and dietary habits, general health status, medication use, and sampling season [1]. Body hair morphology has already been studied in males [52], whilst female scalp hair may be longer for longitudinal mapping. Sampling was done in such a manner to minimize elemental contamination using ceramic scissors [55]. Analysis of the body hair was performed on individual hairs for which the same regional areas of fresh hairs were analyzed for each respective technique.

### 3.2. Preparation of Samples for SR-XRF Elemental Micro-Mapping

To preserve metals deposited on the hair surface and avoid redistribution of elements within the hair matrix for longitudinal mapping, unwashed hair samples were used [1]. Since the X-rays’ fluorescence photons reach the detector depending on the escape depth of the sample, thin cross-sections of the hairs were prepared for cross-sectional analysis [56]. For this purpose, individual hairs were embedded in paraffin wax and sectioned with a microtome to a thickness of 10 µm. Cross-sectioned hairs were mounted on ultralene films, whilst intact hair sections for longitudinal mapping were affixed to sample holders, photographed with a light microscope, and introduced into the TwinMic low energy SR-XRF experimental setup for longitudinal and transverse elemental micro-mapping.

### 3.3. SR-XRF Elemental Micro-Mapping

Specimens were analyzed using a TwinMic microscope in scanning mode [57,58] at an energy of 2 keV in order to excite Si, Al, Mg, Na, Fe, O and possibly As. The X-ray beam was focused on the sample plane through Au zone plate diffractive optics with a diameter of 600 µm and an outermost zone width of 50 nm. For the present experiment, a 500 nm X-ray beam spot size was chosen, which was a good compromise between the signal and the size of the features of interest. A fast readout CCD camera collected the transmitted X-rays, producing absorption and differential phase contrast images [59] of the analyzed areas, while the X-ray fluorescence intensity was measured by eight Si-drift detectors concentrically mounted at a 20 grazing angle with respect to the specimen plane and at a detector-to-specimen distance of 28 mm [60]. The zone plate, sample, and detectors were all placed under vacuum (10^−6^ mbar pressure), thus avoiding any absorption and scattering by air. The acquired XRF spectra were deconvolved and analyzed using PyMCA software (version 5.3.1, European Synchrotron Radiation Facility, Grenoble, France) [61].

### 3.4. SEM-EDX

Samples were carbon coated with an evaporation coater (MED 010, Balzers Union, Liechtenstein), and the morphology of samples was assessed using SEM with a Tescan MIRA3. Sample elemental composition was determined with EDX using a Nova NanoSEM equipped (Oxford Instruments, Oxfordshire, United Kingdom) with an Oxford X-Max detector operating at 20 kV (20 mm^2^, 6 mm working distance) and Oxford INCA software (version 1.1.0.34, Oxford Instruments, Abingdon, United Kingdom). Morphometric analysis of SEM images was performed using the free software ImageJ [version 1.8, National Institutes of Health, Bethesda, MD, USA, (http://rsbweb.nih.gov/ij/), accessed 1 January 2022], which enables the conversion of pixels to units of measure [62]. The scale layer difference was measured that represents the average value of ca. ten distances between two adjacent scales on the same hair [53]. The data were presented as SEM-EDX images, mean ± SD, and a spider graph.

### 3.5. ATR-FTIR

The biochemical profile of intact hair fibers was investigated using ATR-FTIR carried out using a Thermo Scientific Nicolet iS10 Spectrometer (Thermo Scientific, Waltham, MA, USA) equipped with a Smart iTR ATR accessory with a diamond crystal. ATR-FTIR absorption spectra were obtained in the range of 4000–60 cm^−1^ using 64 scans at a resolution of 4 cm^−1^. The spectra were collected at room temperature using Thermo Scientific OMNIC software (version 9.2.41, Thermo Fisher Scientific OMNIC, Waltham, MA, USA).

### 3.6. DSC

DSC was carried out using a TA Instruments Q200 instrument (TA Instruments, New Castle, DE, USA). The sample (2–5 mg) was placed in a sealed Al pan, and the change in the heat flow of the sample was recorded as a function of temperature relative to an empty sealed reference pan. Any energetic event was recorded as an endothermic or exothermic peak relative to temperature. The temperature was ramped between 253 and 573 K using a rate of 5–10 K min^−1^, depending on the sample. Dry nitrogen gas was used to purge the furnace at a flow rate of 50 mL min^−1^. Universal Analysis 2000 software (version 4.5.0.5, TA Instruments, New Castle, DE, USA) was used to analyze the data.

### 3.7. Statistical Analyses

For SEM and EDX data, the data were summarized using summative statistics and an empirical approach used for DSC data. For the ATR-FTIR biochemical and other quantitative data, PCA was used as a chemometric analysis tool, whilst multivariate analysis was performed on EDX elemental data using heatmap analysis. A heatmap was constructed for the median profiles in clusters using the heatmap function in the R Statistical Software (version 3.4.2, RStudio Inc., Boston, MA, USA) and served as both a visualization and partitioning tool. Dendrograms were added to the margins of the heatmap using hierarchical clustering with a complete linkage method based on the Euclidean distances among clusters in rows or among elements in columns. The experimental data were standardized and scaled prior to heatmap analysis as wide differences in data dimensionality may lead to misclassification [63].

## 4. Conclusions

Although human hair has been studied from multiple perspectives by anthropologists, biologists, geneticists, forensic scientists, and cosmetic scientists, understanding of differences between body hairs has been extremely limited. This study presents the first case report with control for external confounding variables to explore the utility of a multidimensional qualitative and semi-quantitative approach using both synchrotron (SR-XRF for longitudinal and hair morphological region mapping) and benchtop methods including, ATR-FTIR (supported by chemometrics analysis with PCA), DSC, SEM, and EDX (supported by heatmap analysis) to screen different body hairs in terms of its elemental, biochemical, thermal, and cuticle properties. For SEM and DSC, only scalp hairs sampled from the nape of the neck area were used that showed a well-preserved physico-chemical makeup. Based on the elemental mapping of elements across the different body hairs, hair ends, eyebrow and leg hair had the most dissimilar elemental distribution patterns compared to other body hairs. This observation in terms of thermal properties was also noted for DSC analysis, whilst hair ends, eyebrow and leg hair were also grouped together in the PCA analysis based on hair biochemical content. Scale layer differences reflective of the hair growth cycle were also similar for eyebrow and leg hair. The intricate and rather complex interplay between the organization and levels of elements and biomolecules within the crystalline and amorphous matrix of different hairs that also contribute to their structural integrity is highlighted by these results. It should further be noted that external factors, as affected by a locality, may also significantly affect the physico-chemical profile of different body hairs. In conclusion, hair chemistry linked to its material properties (i.e., morphometry and thermal profile) remains complex and varied as influenced by multifactorial factors, a finding that may have important implications for forensic science, toxicology and systemic intoxication, or other studies involving hair as a research matrix. A limitation of this study was, however, that low energy SR-XRF did not detect a wider range of metals in the hair sample and in the future, such multidimensional analysis should be complemented with quantitative elemental mapping using proton-induced X-ray emission analysis [2,64] or XRF in the hard X-ray regime. Future fundamental studies controlling for interlaboratory reliability should also assess differences between the physico-chemical properties of (1) various points along the hair length between different body hairs; (2) body hairs that include the hair bulb unexposed to the external environment; (3) body hairs representing different genders; and (4) body hairs representing regional populations.

## Data Availability

Data are contained within the article.

## Supplementary Materials

The supporting information can be downloaded at: https://www.mdpi.com/article/10.3390/ijms24044166/s1.

## References

[1] Kempson I.M., Lombi E. (2011). Hair analysis as a biomonitor for toxicology, disease and health status. Chem. Soc. Rev..

[2] Kučera J., Kameník J., Havránek V. (2018). Hair elemental analysis for forensic science using nuclear and related analytical methods. Forensic Chem..

[3] Bertrand L., Vichi A., Doucet J., Walter P., Blanchard P. (2014). The fate of archaeological keratin fibres in a temperate burial context: Microtaphonomy study of hairs from Marie de Bretagne (15th c., Orléans, France). J. Archaeol. Sci..

[4] Cloete K.J. (2018). Ion Beam, Synchrotron Radiation, and Related Techniques in Biomedicine: Elemental Profiling of Hair.

[5] Usman M., Naseer A., Baig Y., Jamsheid T., Shahwar M., Khurshuid S. (2019). Forensic toxicological analysis of hair: A review. Egypt. J. Forensic Sci..

[6] Popescu C., Höcker H. (2007). Hair—The most sophisticated biological composite material. Chem Soc. Rev..

[7] Robbins C.R., Robbens C.R. (2012). Chemical Composition of Different Hair Types. Chemical and Physical Behavior of Human Hair.

[8] Dahiya M.S., Yadav S.K. (2013). Elemental Composition of Hair and its Role in Forensic Identification. CiteseerX.

[9] Lorentz K.O., de Nolf W., Cotte M., Ioannou G., Foruzanfar F., Zaruri M.R., Sajjadi S.M.S. (2020). Synchrotron radiation micro-X-Ray Fluorescence (SR-μXRF) elemental mapping of ancient hair: Metals and health at 3rd millennium BCE Shahr-i Sokhta, Iran. J. Archaeol. Sci..

[10] Israelsson A., Eriksson M., Pettersson H.B.L. (2015). On the distribution of uranium in hair: Non-destructive analysis using synchrotron radiation induced X-ray fluorescence microprobe techniques. Spectrochim. Acta Part B At. Spectrosc..

[11] Martin R.R., Kempson I.M., Naftel S.J., Skinner W.M. (2005). Preliminary synchrotron analysis of lead in hair from a lead smelter worker. Chemosphere.

[12] Lum J.T.-S., Chan Y.-N., Leung K. (2021). S-Y. Current applications and future perspectives on elemental analysis of non-invasive samples for human biomonitoring. Talanta.

[13] Alkhuder K. (2022). Attenuated total reflection-Fourier transform infrared spectroscopy: A universal analytical technique with promising applications in forensic analyses. Int. J. Legal Med..

[14] Smith B.C. (1998). Infrared Spectral Interpretation: A Systematic Approach.

[15] Skoog D.A., James Holler F., Crouch S.R. (2007). Principles of Instrumental Analysis.

[16] Gandhi K., Sharma N., Gautam P.B., Sharma R., Mann B., Pandey V., Gandhi K., Sharma N., Gautam P.B., Sharma R., Mann B., Pandey V. (2022). Infrared (IR) Spectroscopy. Advanced Analytical Techniques in Dairy Chemistry.

[17] Grdadolnik J. (2003). Saturation effects in FTIR spectroscopy: Intensity of Amide I and Amide II bands in protein spectra. Acta. Chim. Slov..

[18] Yu Y., Yang W., Wang B., Meyers M.A. (2017). Structure and mechanical behavior of human hair. Mater. Sci. Eng. C.

[19] De Castro Lima C.R.R., Machado L.D.B., Velasco M.V.R., Mator J.d.R. (2018). DSC measurements applied to hair studies. J. Therm. Anal. Calorim..

[20] Rendón-Lugo A.N., Santiago P., Puente-Lee I., León-Paniagua L. (2017). Permeability of hair to cadmium, copper and lead in five species of terrestrial mammals and implications in biomonitoring. Environ. Monit. Assess..

[21] Ikarashi Y., Uchino T., Nishimura T. (2010). Analysis of preservatives used in cosmetic products: Salicylic acid, sodium benzoate, sodium dehydroacetate, potassium sorbate, phenoxyethanol, and parabens. Bull. Natl. Inst. Health Sci..

[22] Allison A., Fouladkhah A. (2018). Adoptable interventions, human health, and food safety considerations for reducing sodium content of processed food products. Foods.

[23] Pirovano P., Dorrian M., Shinde A., Donohoe A., Brady A.J., Moyna N.M., Wallace G., Diamond D., McCaul M. (2020). A wearable sensor for the detection of sodium and potassium in human sweat during exercise. Talanta.

[24] Chen Y.-L., Kuan W.-H., Liu C.-L. (2020). Comparative study of the composition of sweat from eccrine and apocrine sweat glands during exercise and in heat. Int. J. Environ. Res. Public Health.

[25] Abrar M., Iqbal T., Fahad M., Andleeb M., Farooq Z., Afsheen S. (2018). Determination of hazardous ingredients in personal care products using laser-induced breakdown spectroscopy. Laser Phys..

[26] Li M., Liu Z., Chen Y., Zhang M. (2019). Identifying effects of pipe material, hydraulic condition, and water composition on elemental accumulation in pipe corrosion scales. Environ. Sci. Pollut. Res..

[27] Alasfar R.H., Isaifan R.J. (2021). Aluminum environmental pollution: The silent killer. Environ. Sci. Pollut. Res..

[28] Kempson I.M., Henry D.A. (2010). Determination of arsenic poisoning and metabolism in hair by synchrotron radiation: The case of Phar Lap. Angew. Chem. Int. Ed. Engl..

[29] Bates R.A., Salsberry P.J., Ford J.L., Pickler R.H., Dynia J.M., Justice L.M. (2020). Hair sampling for cortisol analysis with mother-toddler dyads living in low-income homes. Infant Behav. Dev..

[30] Vladmir B. (1995). Use of human hair as a biomarker in the assessment of exposure to pollutants in occupational and environmental settings. Toxicology.

[31] Hong L., Simon J.D. (2007). Current understanding of the binding sites, capacity, affinity, and biological significance of metals in melanin. J. Phys. Chem. B.

[32] Bolduc C., Shapiro J. (2001). Hair care products: Waving, straightening, conditioning, and coloring. Clin. Dermatol..

[33] Blume-Peytavi U., Tosti A., Trüeb R.M., Whittig D.A. (2008). Hair Growth and Disorders.

[34] Rawat K., Sharma N., Singh V., Singh V.K., Kawai J., Tripathi D.K. (2022). X-Ray Fluorescence and comparison with other analytical methods (AAS, ICP-AES, LA-ICP-MS, IC, LIBS, SEM-EDS, and XRD). X-ray Fluorescence in Biological Sciences: Principles, Instrumentation, and Applications.

[35] Sundaram K., Gunasekaran S., Sailatha E., Marthandam P., Kuppuraj P. (2016). FTIR-ATR spectroscopic technique on human single intact hair fibre -A case study of thyroid patients. Int. J. Adv. Sci. Technol. Eng. Manag. Sci..

[36] Prompong P., Chuchaat T., Suwimol N., Sanong E. (2018). Analysis of cosmetic residues on a single human hair by ATR FT-IR microspectroscopy. Spectrochim. Acta Part A Mol. Biomol. Spectrosc..

[37] Shan-Yang L., Me-Jane L., Wen-Ting C. (2007). FT-IR and Raman vibrational microspectroscopies used for spectral biodiagnosis of human tissues. J. Spectrosc..

[38] Kumar R., Sharma V. (2018). Chemometrics in forensic science. Trends Anal. Chem..

[39] Manheim J., Doty K.C., McLaughlin G., Lednev I.K. (2016). Forensic hair differentiation using Attenuated Total Reflection Fourier Transform Infrared (ATR FT-IR) Spectroscopy. Appl. Spectrosc..

[40] Monteiro V., Maciel A., Longo E. (2005). Thermal analysis of Caucasian human hair. J. Therm. Anal. Calorim..

[41] Popescu C., Gummer C. (2016). DSC of human hair: A tool for claim support or incorrect data analysis?. Int. J. Cosmet. Sci..

[42] Lim Y.S., Harland D.P., Dawson T.L. (2019). Wanted, dead and alive: Why a multidisciplinary approach is needed to unlock hair treatment potential. Exp. Dermatol..

[43] Wortmann F.J., Wortmann G., Popescu C. (2020). Linear and nonlinear relations between DSC parameters and elastic moduli for chemically and thermally treated human hair. J. Therm. Anal. Calorim..

[44] Ionashiro E., Hewer T., Fertonani F., Almeida E., Ionashiro M. (2003). Application of differential scanning calorimetry in hair samples as a possible tool in Forensic Science. Eclét. Quím. J..

[45] Sanchita M., Jyoti R.G., Arup R.B. (2010). Histomorphological and quantitative characteristics of black and gray human scalp hair. J. Life Sci..

[46] Swift J., Smith J. (2006). Atomic force microscopy of human hair. Scanning.

[47] Koch S.L., Tridico S.R., Bernard B.A., Shriver M.D., Jablonski N.G. (2020). The biology of human hair: A multidisciplinary review. Am. J. Hum. Biol..

[48] Tung J.K., Yasuda M.R., Lee L.N. (2020). Anatomy and Physiology of the Hair Cycle. Hair Transplant Surgery and Platelet Rich Plasma.

[49] Ahmed Y.A., Ali S., Ghallab A. (2018). Hair histology as a tool for forensic identification of some domestic animal species. EXCLI J..

[50] Felix G.A., Soares Fioravanti M.C., Cassandro M., Tormen N., Quadros J., Juliano R.S., Alves do Egito A., Ivete de Moura M., Piovezan U. (2019). Bovine breeds identification by trichological analysis. Animals.

[51] Madkour F.A., Abdelsabour-Khalaf M. (2022). Performance scanning electron microscopic investigations and elemental analysis of hair of the different animal species for forensic identification. Microsc. Res. Tech..

[52] Hess W.M., Seegmiller R.E., Gardner J.S., Allen J.V., Barendregt S. (1990). Human hair morphology: A scanning electron microscopy study on a male Caucasoid and a computerized classification of regional differences. Scanning Microsc..

[53] Lee L.C., Wan Mohamad Fuad W.N.S., Abdullah S.S., Ong K.L. (2019). Preliminary study on morphometric analysis of the human scalp hair for discrimination of ethnic Malay and ethnic Chinese in Malaysia. Egypt. J. Forensic Sci..

[54] Kataria S., Dabas P., Saraswathy K.N., Sachdeva M.P., Jain S. (2023). Investigating the morphology and genetics of scalp and facial hair characteristics for phenotype prediction. Sci. Justice.

[55] Anderson H., Morris J. (1992). Contamination studies in the use of human nails for dietary studies: The effect of clippers. J. Radioanal. Nucl. Chem..

[56] Martin R.R., Naftel S.J., Nelson A.J., Feilen A.B., Narvaez A. (2004). Synchrotron X-ray fluorescence and trace metals in the cementum rings of human teeth. J. Environ. Monit..

[57] Gianoncelli A., Kourousias G., Merolle L., Altissimo M., Bianco A. (2016). Current status of the TwinMic beamline at Elettra: A soft X-ray transmission and emission microscopy station. J. Synchrotron Radiat..

[58] Gianoncelli A., Bonanni V., Gariani G., Guzzi F., Pascolo L., Borghes R., Billè F., Kourousias G. (2021). Soft X-ray microscopy techniques for medical and biological imaging at TwinMic—Elettra. Appl. Sci..

[59] Gianoncelli A., Morrison G.R., Kaulich B., Bacescu D., Kovac J. (2006). Scanning transmission x-ray microscopy with a configurable detector. Appl. Phys. Lett..

[60] Gianoncelli A., Kourousias G., Stolfa A., Kaulich B. (2013). Recent developments at the TwinMic beamline at ELETTRA: An 8 SDD detector setup for low energy X-ray. J. Phys. Conf. Ser..

[61] Solé V., Papillon E., Cotte M., Walter P., Susini J. (2007). A multiplatform code for the analysis of Energy-Dispersive X-ray Fluorescence Spectra. Spectrochim. Acta Part B Spectrosc..

[62] Crawford E.C., Mortensen J.K. (2009). An ImageJ11ImageJ: http://rsb.info.nih.gov/ij/. plugin for the rapid morphological characterization of separated particles and an initial application to placer gold analysis. Comput. Geosci..

[63] Liu C.W., Lin K.H., Kuo Y.M. (2003). Application of factor analysis in the assessment of groundwater quality in a Blackfoot disease area in Taiwan. Sci. Total Environ..

[64] Dang Z., Yu T., Xu H., Zhang H., Ren Q., Shen H. (2020). Investigation on the 2D-distribution of metallic elements after hair dyeing. Biol. Trace Elem. Res..

