# Supplementary material for: Multidimensional Profiling of Human Body Hairs Using Qualitative and Semi-Quantitative Approaches with SR-XRF, ATR-FTIR, DSC, and SEM-EDX
Source: Int J Mol Sci. 2023 Feb 19;24(4):4166. doi: 10.3390/ijms24044166 (PMC9964782; doi:10.3390/ijms24044166)
Supplement: Supplementary file 1 [file ijms-24-04166-s001.zip › ijms-2148041-supplementary.pdf]

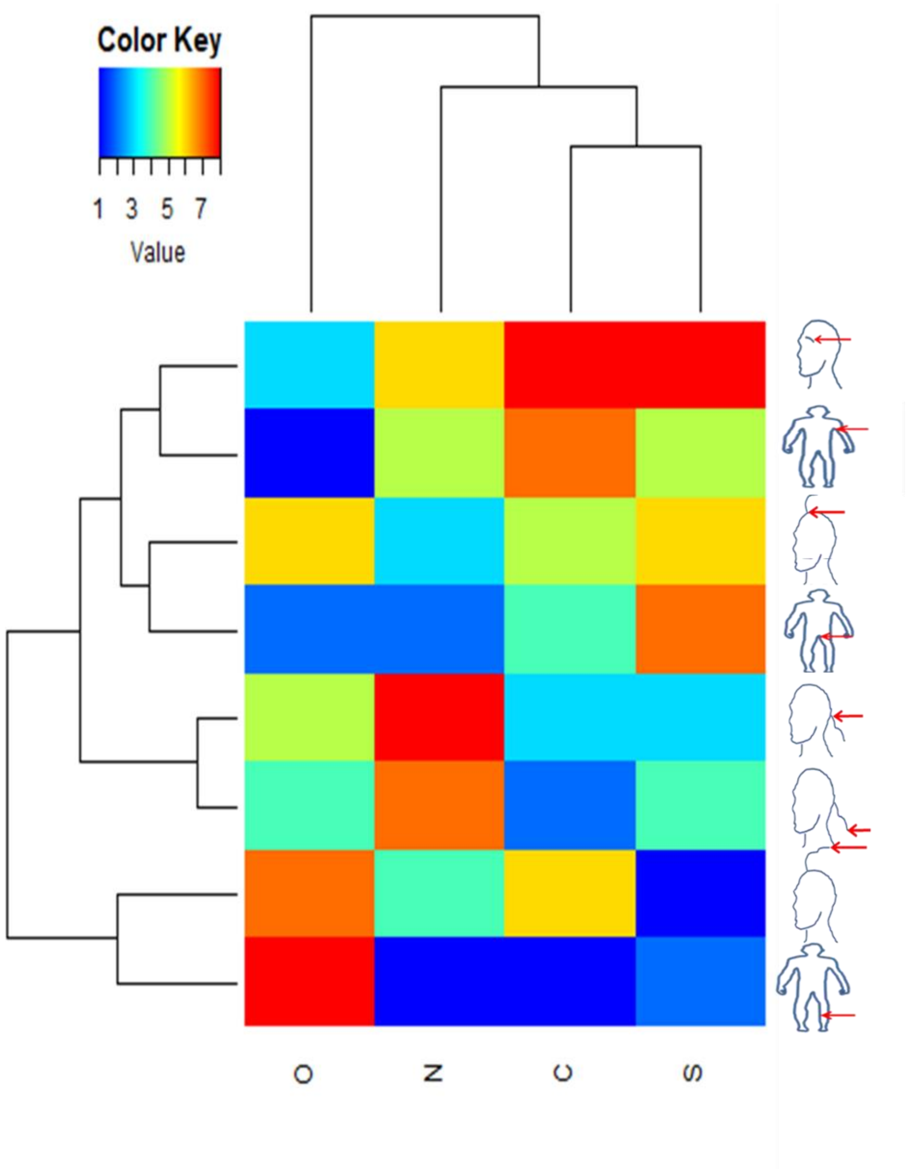

Figure S1. Heatmap generated using complete linkage hierarchical clustering based on Euclidean distances showing the median elemental abundances (quantified as moderated z-scores) of clusters for elemental compositions of body hair from different regions. The relationship is presented by a dendrogram in which rows represent body region and columns elements, with a specific color representing the magnitude of abundance (please refer to the text for a full explanation of the heatmap results).
